# Procollagen-lysine, 2-oxoglutarate 5-dioxygenases 1, 2, and 3 are potential prognostic indicators in patients with clear cell renal cell carcinoma

**DOI:** 10.18632/aging.102206

**Published:** 2019-08-25

**Authors:** Wen-Hao Xu, Yue Xu, Jun Wang, Xi Tian, Junlong Wu, Fang-Ning Wan, Hong-Kai Wang, Yuan-Yuan Qu, Hai-Liang Zhang, Ding-Wei Ye

**Affiliations:** 1 Department of Urology, Fudan University Shanghai Cancer Center, Shanghai 200032, P.R. China; 2 Department of Oncology, Shanghai Medical College, Fudan University, Shanghai 200032, P.R. China; 3 Department of Ophthalmology, The First Affiliated Hospital of Soochow University, Suzhou 215000, P.R. China

**Keywords:** aging, lymphatic vessels, endothelial cells

## Abstract

Intratumoral fibrosis is a frequent histologic finding in highly vascularized clear cell renal cell carcinoma (ccRCC). Here, we investigated the expression of a family of collagen-modifying enzymes, procollagen-lysine, 2-oxoglutarate 5-dioxygenases 1, 2, and 3 (PLOD1/2/3), in ccRCC tissues and assessed the prognostic value of wild-type and genetically mutated PLOD1/2/3 for ccRCC patients. Normal kidney and ccRCC mRNA and protein expression datasets were obtained from Oncomine, The Cancer Genome Atlas, and Human Protein Atlas databases. Associations between PLOD1/2/3 expression, clinicopathological variables, and patient survival were evaluated using Cox regression and Kaplan–Meier analyses. PLOD1/2/3 mRNA and protein expression levels were significantly elevated in ccRCC tissues compared with normal kidney. Increased PLOD1/2/3 mRNA expression was significantly associated with advanced tumor stage, high pathological grade, and shorter progression-free and overall survival (all *p*<0.01). Genetic mutation of PLOD1/2/3 was present in ~3% of ccRCC patients and was associated with significantly poorer prognosis compared with expression of wild-type PLOD1/2/3 (*p*<0.001). This study thus identifies tumor expression of wild-type or mutated PLOD1/2/3 mRNA as a potential predictive biomarker for ccRCC patients and sheds light on the underlying molecular pathogenesis of ccRCC.

## INTRODUCTION

Renal cell carcinoma (RCC) is one of the most common malignancies of the genitourinary system and accounts for approximately 3% of malignant tumors in adults and 2% of all cancer deaths [1]. Worldwide morbidity and mortality rates from RCC are increasing at approximately 2% to 3% per decade [2]; however, the rates have stabilized or declined in many developed countries over the past several years [3]. Clear cell RCC (ccRCC) is the most common histological subtype of RCC, accounting for about 70% of cases. The incidence of ccRCC is affected by demographic and geographic factors, and, to a lesser extent, hereditary factors. Accumulating studies have been done on understanding the biological heterogeneity of ccRCC and its aggressive behaviors. Therefore, there is an urgent need to better understand the molecular alterations involved in the initiation and progression of ccRCC to assist in the development of novel prognostic markers and more effective treatment regimens.

Collagen, which occurs in at least tissue-dependent forms, is the major component of the extracellular matrix. Interactions between collagen and several cell surface receptors activates biochemical and biophysical signaling pathways that are crucial for normal cellular functions, including cell movement, proliferation, and survival. Thus, it is not surprising that mutations in collagen and/or defects in its synthesis, processing, and assembly may contribute to the development and progression of cancer [4, 5].

Hydroxylation of lysyl residues is one of the key steps in collagen biosynthesis, and usually occurs at the Y position in the repeating glycine-X-Y motif [6, 7]. Collagen cross-linking and deposition depend on lysyl hydroxylation, which is catalyzed by prolyl 4-hydroxylase and procollagen-lysine, 2-oxoglutarate 5-dioxygenase (PLOD). The three PLOD isoforms identified to date (PLOD1, PLOD2, and PLOD3 [8–10]), have similar primary structures and share 47% similarity at the amino acid sequence level [6]. PLOD1/2/3 expression is mainly regulated through transcription and is modulated by the activity of various transcription factors (e.g., hypoxia inducible factor-1α [HIF-1α] and E2Fs), cytokines (e.g., transforming growth factor-β [TGF-β]), and growth factors (e.g., bone morphogenetic protein-2 [BMP-2]) [11–13]. Previous studies have implicated mutation or overexpression of PLODs in many human diseases, including neoplasms such as breast, colorectal, and lung carcinomas [14–16] and Ehlers–Danlos syndrome, an autosomal recessive disorder characterized by joint and skin abnormalities [17]. Excessive collagen cross-linking has also been associated with the pathogenesis of irreversible, fibrotic kidney lesions [18]. Intratumoral fibrosis is a frequent histologic finding in solid organ tumors, including lung cancer, breast cancer, and highly vascularized RCC [19, 20]. Previous work has shown that intratumoral fibrosis is associated with several indicators of poor prognosis in ccRCC, such as Fuhrman nuclear grade and lymphovascular invasion [21]. Therefore, understanding the regulation of expression and function of PLODs in normal organ development and disease progression may identify potential targets for the treatment of diseases induced by aberrant collagen regulation.

In this study, we analyzed publicly available gene and protein expression datasets to investigate the differential expression of PLOD1/2/3 at the mRNA and protein levels and to define their biological interaction networks in ccRCC. We also assessed the genetic mutation rate of the three isoforms and determined the prognostic value of wild-type and mutated PLOD1/2/3 in ccRCC. The goal of the study was to provide insights into the molecular mechanisms of ccRCC and to reveal potential novel therapeutic targets for this disease.

## RESULTS

### Elevated expression of PLOD1/2/3 in ccRCC

**Figure 1:**
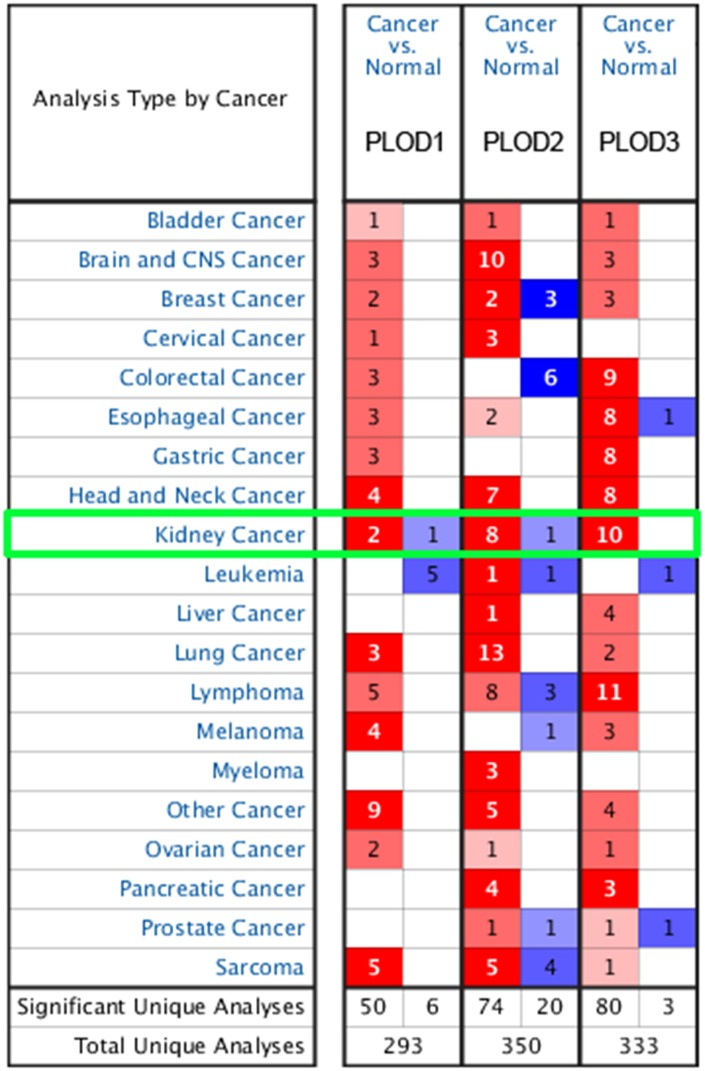
**Transcriptional expression of PLODs in 20 different types of cancer diseases from Oncomine database.** Difference of transcriptional expression was compared by Students’ t-test. Cut-off of *p* value and fold change were as following: *p* value=0.01, Fold Change=1.5, gene rank=10%, data type: mRNA.

To begin to assess the potential involvement of PLODs in ccRCC, we first analyzed their differential expression at the mRNA and protein level using multiple datasets hosted on the Oncomine, TCGA, and Human Protein Atlas platforms. As shown in Figure 1, analysis of PLOD1, PLOD2, and PLOD3 mRNA expression in 20 cancer types (Oncomine dataset) showed significant upregulation and downregulation of the three PLOD isoforms in a number of cancers, including kidney cancer, compared with the corresponding normal tissues. PLOD mRNA levels were significantly elevated in ccRCC compared with normal kidney in multiple datasets totaling 153 clinical specimens and 71 normal kidney samples (referred to as Yusenko [22], Jones [23], Beroukhim [24], Gumz [25], Higgins [26], and Lenburg [27] datasets: Table 1). In the Yusenko Renal dataset (n=31), PLOD1, 2, and 3 mRNA levels were elevated in ccRCC tissue compared with normal tissue by 2.365-fold (*p*=1.04^−14^), 5.425-fold (*p*=6.93^−05^), and 2.766-fold (*p*=2.57^−09^), respectively. In the Jones Renal dataset (n=46), the fold increases in PLOD1, 2, and 3 mRNA were 2.683 (*p*=2.17^−09^), 5.761 (*p*=1.75^−15^) and 2.200 (*p*=5.36^−12^), respectively. In the Beroukhim Renal dataset, PLOD2 and PLOD3 were significantly overexpressed in non-hereditary ccRCC tissues (5.512-fold, *p*=6.57^−12^ and 1.719, *p*=3.59^−08^, respectively) (n=38) and in hereditary ccRCC tissues (6.562-fold, *p*=4.15^−13^ and 2.007, *p*=3.75^−10^, respectively) (n=43) compared with normal tissues. In the Gumz Renal dataset (n=20), significant amplification of PLOD2 was detected in ccRCC tissues compared with normal tissues (4.249-fold, *p*=4.65^−08^), and similarly, the Higgins Renal dataset (n=29) showed a 4.721-fold increase in PLOD2 (*p*=4.89^−05^) and 2.322-fold increase in PLOD3 mRNA (*p*=2.00^−03^) in ccRCC samples compared with normal kidney tissue. Finally, the Lenburg Renal dataset (n=18) also demonstrated upregulated levels of PLOD2 mRNA (3.945-fold, *p*=1.00^−03^) and PLOD3 mRNA (1.928-fold, *p*=9.18^−06^).

**Table 1:** Significant changes of PLODs expression in transcription level between kidney renal clear cell carcinoma (KIRC) and normal renal tissues (ONCOMINE).

	**Types of KIRC VS. Kidney**	**Fold Change**	***p* value**	**t-test**	**Ref.**
PLOD1					
	Clear Cell Renal Cell Carcinoma	2.365	1.04E-14	13.993	Yusenko Renal
	Clear Cell Renal Cell Carcinoma	2.683	2.17E-09	8.493	Jones Renal
PLOD2					
	Non-Hereditary Clear Cell Renal Cell Carcinoma	6.562	4.15E-13	11.156	Beroukhim Renal
	Hereditary Clear Cell Renal Cell Carcinoma	5.512	6.57E-12	12.864	Beroukhim Renal
	Clear Cell Renal Cell Carcinoma	4.249	4.65E-08	8.560	Gumz Renal
	Clear Cell Renal Cell Carcinoma	4.721	4.89E-05	7.104	Higgins Renal
	Clear Cell Renal Cell Carcinoma	5.761	1.75E-15	12.781	Jones Renal
	Clear Cell Renal Cell Carcinoma	5.425	6.93E-05	7.026	Yusenko Renal
	Clear Cell Renal Cell Carcinoma	3.945	1.00E-03	3.534	Lenburg Renal
PLOD3					
	Clear Cell Renal Cell Carcinoma	2.766	2.57E-09	11.058	Yusenko Renal
	Clear Cell Renal Cell Carcinoma	2.322	2.00E-03	7.268	Higgins Renal
	Clear Cell Renal Cell Carcinoma	1.928	9.18E-06	7.268	Lenburg Renal
	Non-Hereditary Clear Cell Renal Cell Carcinoma	2.007	3.75E-10	8.847	Beroukhim Renal
	Hereditary Clear Cell Renal Cell Carcinoma	1.719	3.59E-08	8.129	Beroukhim Renal
	Clear Cell Renal Cell Carcinoma	2.200	5.36E-12	10.631	Jones Renal

**Figure 2:**
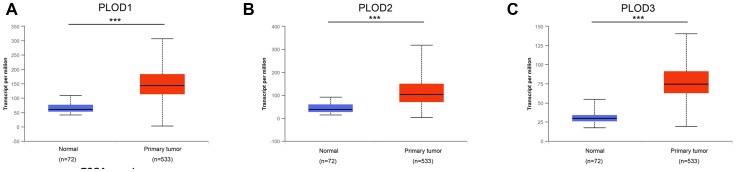
**Transcriptional expression of distinct PLODs family members in ccRCC tumor tissues and adjacent normal renal tissues.** (**A**–**C**) Differential mRNA expressions of 3 PLODs family members were demonstrated to be highly expressed in primary tumor tissues compared to normal samples (*** *p*<0.001).

Analysis of PLOD1/2/3 mRNA expression from the TCGA RNA-sequence database revealed a similar pattern of overexpression in 533 ccRCC tissues compared with 72 normal tissues (*p*<0.001; Figure 2A–2C). In addition, PLOD1/2/3 mRNA expression profiles of multi-tumors and corresponding normal tissues were illustrated in Supplementary Figure 1.

**Figure 3:**
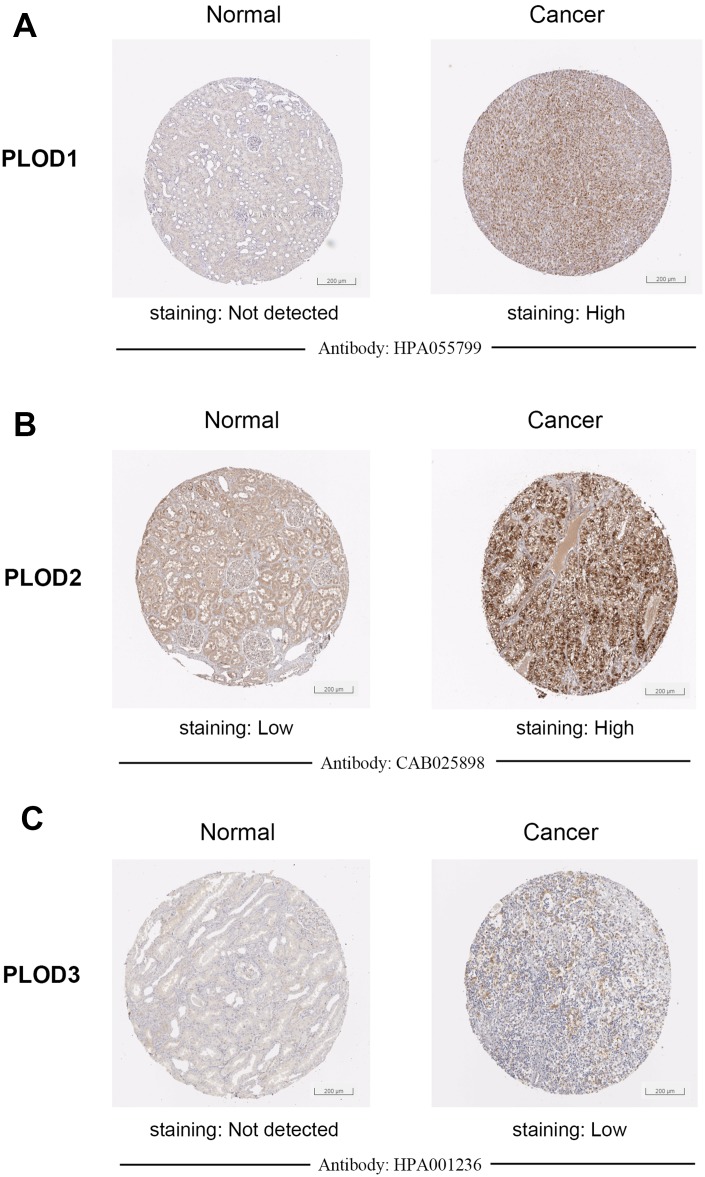
**Representative proteins expressions of IHC images of distinct PLODs family members were detected in ccRCC and normal tissues (Human Protein Atlas).** (**A**–**C**) PLOD1/3 proteins were found not expressed in normal renal tissues, while significantly high staining expressions were observed in ccRCC tissues. (**B**) PLOD2 protein was detected low expressed in normal renal tissues, while high protein expressions were observed in ccRCC tissues.

To verify the transcriptional profiles, we examined PLOD1/2/3 protein expression in ccRCC and normal tissues using staining and expression data obtained from the Human Protein Atlas platform. Representative images of immunohistochemically stained tissues indicated that PLOD1 and PLOD3 proteins were virtually undetectable in normal kidney but were present at very high levels in ccRCC tissues (Figure 3A–3C). In contrast, PLOD2 protein was present in both normal and ccRCC tissues, but the expression level was much higher in the tumor sections (Figure 3B). Taken together, these results indicate that PLOD1, 2, and 3 are transcriptionally and translationally upregulated in ccRCC.

### Association of PLOD1/2/3 mRNA expression and clinicopathological parameters in ccRCC

**Figure 4:**
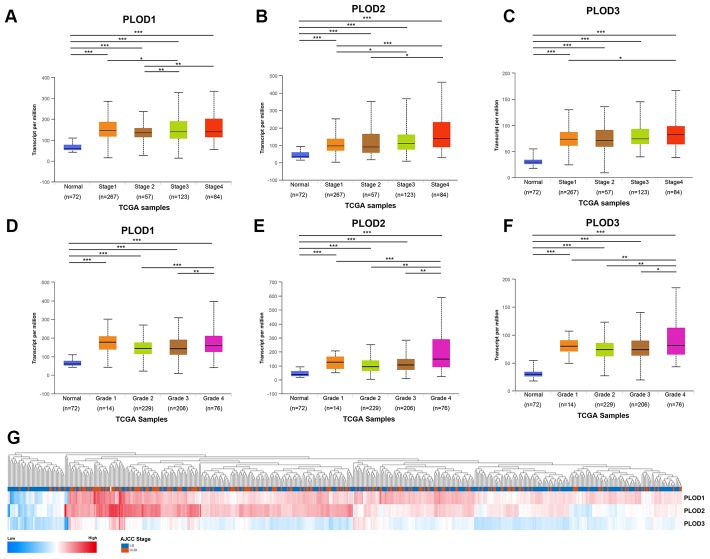
**Relationship between transcriptional expressions of distinct PLODs family members and individual cancer stages and pathological grade of ccRCC patients.** (**A**–**C**) Transcriptional expressions of PLOD1, PLOD2 and PLOD3 were significantly correlated with individual cancer stages, patients who were in more advanced stages tended to express higher mRNA expression of PLODs. (**D**–**F**) Transcriptional expressions of PLOD1, PLOD2 and PLOD3 were significantly correlated with individual pathological grade, patients who were in more advanced grade score tended to express elevated mRNA expression of PLODs. The highest mRNA expressions of PLODs were found in stage 4 or grade 4. *p<0.05, **p<0.01, ***p<0.001. (**G**) Hierarchical partitioning was performed using transcriptional expression profiles of PLODs in a heat map. Color gradients suggest high (red) or low (blue) expression level, indicating a trend that higher AJCC stage was associated with elevated PLODs expression.

Next, we interrogated the TCGA ccRCC dataset to determine whether PLOD1/2/3 expression was associated with clinicopathological features or patient survival. Notably, we found significant correlations between PLOD1/2/3 mRNA expressions and advancing clinical stages (AJCC 1–4, Figure 4A–4C) and pathological grade (ISUP 1–4, Figure 4D–4F). Consistent with this, PLOD1, 2, and 3 mRNA expressions was highest in stage 4 and grade 4 ccRCC tissues. In addition, hierarchical partitioning confirmed the association between higher AJCC stage and elevated PLOD1/2/3 mRNA expression (Figure 4G). Thus, elevated PLOD expression in ccRCC patients was significantly associated with disease progression.

### Prognostic implications of PLOD1/2/3 mRNA expression in ccRCC

Given the association between PLOD1/2/3 expression and disease stage/grade, we next determined whether we could detect a similar association with patient survival. For this, we downloaded and processed level 3 RNA-sequence data from TCGA and performed Cox regression analyses using univariate and multivariate models. As shown in Supplementary Tables 2–4, we evaluated PLOD1, 2, and 3 mRNA levels and several traditional prognostic factors (TNM stage, AJCC stage, and ISUP grade). Univariate Cox logistic regression analysis indicated PLOD1, 2, 3 significantly correlated with PFS (hazard ratio [HR] 3.508, *p*=0.003; 3.467, *p*<0.001, and 2.555, *p*=0.002, respectively) and OS (HR 1.669, *p*=0.010; 2.089, *p*<0.001; and 1.569, *p*=0.005, respectively) in TCGA cohort. Multivariate analysis showed statistical association between PLOD1, PLOD2, and PLOD3 expressions and PFS (HR 1.716, *p*=0.241; 2.503, *p*=0.014, and 2.032, *p*=0.023, respectively), and OS (HR 1.147, *p*=0.525; 1.825, *p*=0.002; and 1.317, *p*=0.101, respectively; Table 2). AJCC stage (ref. I-II) was significantly associated with prognosis of ccRCC patients (PFS, HR 4.866, *p*=0.009; OS, HR 2.411, *p*=0.006).

**Table 2:** Multivariate Cox logistic regression analysis of PFS and OS in TCGA cohort (PFS: progression-free survival; OS: overall survival; TCGA: the Cancer Genome Atlas)

**Covariates**	**PFS**				**OS**		
	**HR**	**95% CI**	***p* value**		**HR**	**95% CI**	***p* value**
pT stage (ref. T1-T2)	0.578	0.215-1.551	0.277		0.786	0.455-1.359	0.389
pN stage (ref. N0)	0.811	0.354-1.860	0.621		1.416	0.933-2.148	0.102
pM stage (ref. M0)	1.651	0.851-3.203	0.138		1.941	1.343-2.804	**<0.001**
AJCC stage (ref. I-II)	4.866	1.478-16.018	**0.009**		2.411	1.280-4.542	**0.006**
ISUP grade (ref. 1-2)	0.240	1.558-0.744	0.240		1.490	1.030-2.157	**0.034**
PLOD1 expression (ref. low)	1.706	0.699-4.164	0.241		1.147	0.752-1.750	0.525
PLOD2 expression (ref. low)	2.503	1.201-5.216	**0.014**		1.825	1.246-2.673	**0.002**
PLOD3 expression (ref. low)	2.032	1.104-3.737	**0.023**		1.317	0.947-1.831	0.101

**Figure 5:**
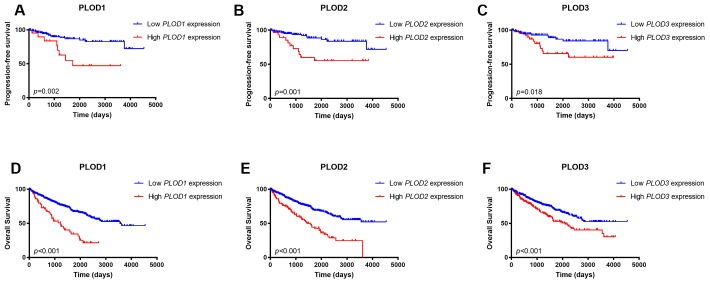
**Kaplan-Meier survival analyses on differential PLOD1/2/3 expression groups with PFS.** (**A**–**C**) and OS (**D**–**F**) in the included 533 ccRCC patients. Compared with low mRNA expression of PLODs, high PLOD1 expressions were significantly correlated with poor PFS (*p*=0.002) and OS (*p*<0.001), high PLOD2 expressions were significantly associated with poor PFS (*p*=0.001) and OS (*p*<0.001), and elevated PLOD3 expressions were significantly correlated with shorter PFS (*p*=0.018) and OS (*p*<0.001).

To confirm these findings, we assigned the 533 patients in the TCGA dataset to two groups based on low or high PLOD1, 2, or 3 mRNA expressions. Kaplan–Meier survival analyses indicated that, for all three PLOD genes, high tumor expression was significantly correlated with shorter PFS (*p*=0.002, *p*=0.001, *p*=0.018, respectively; Figure 5A–5C) and shorter OS (all *p*<0.001; Figure 5D–5F). These data confirm the prognostic value of PLOD1/2/3 expression in ccRCC.

### Genetic mutations in PLOD1/2/3 and their prognostic value in ccRCC

**Figure 6:**
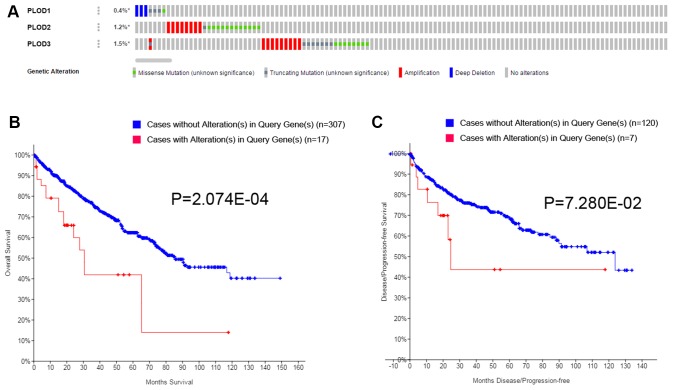
**Genetic mutations in PLODs family members and their association with OS and DFS of ccRCC patients (cBioPortal).** A total of 3.1% mutation rate of PLODs was observed in ccRCC patients. Genetic mutation alterations rate of PLOD1, PLOD2 and PLOD3 were 0.4%, 1.2% and 1.5%, respectively (**A**). Genetic alterations in PLODs were associated with shorter OS (**B**) and DFS (**C**) of ccRCC patients.

Having established the survival implications of wild-type PLOD1/2/3, we next investigated whether the PLOD genes are mutated in ccRCC and, if so, whether mutations influence the prognostic value. For this, we analyzed the TCGA dataset using GISTIC in cBioPortal. As shown in Figure 6, the overall total mutation rate (missense mutations, truncations, amplifications, and deletions) of PLOD1/2/3 in ccRCC patients was 3.1%, with individual rates for PLOD1, PLOD2, and PLOD3 of 0.4%, 1.2%, and 1.5%, respectively (Figure 6A). Kaplan–Meier analysis of the survival of ccRCC patients with or without PLOD1/2/3 mutations demonstrated that both OS (Figure 6B) and PFS (Figure 6C) were significantly shorter for patients expressing mutated compared with wild-type PLOD1/2/3. Thus, the survival of ccRCC patients is influenced not only by the expression level of PLOD1/2/3 but also whether the wild-type *vs* mutated forms are expressed.

### Predicted interaction networks and signaling pathways of PLOD1/2/3

**Figure 7:**
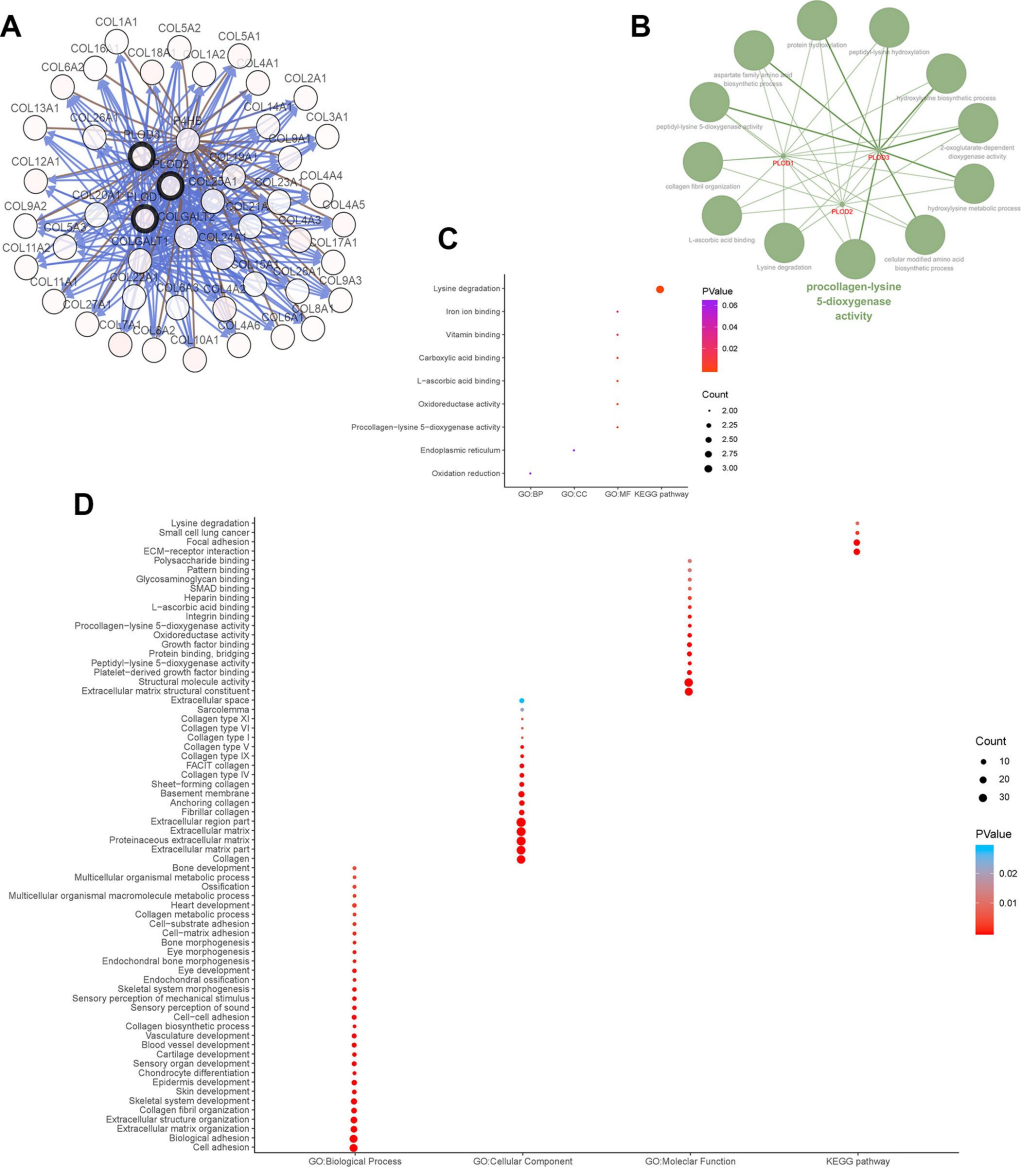
**Functions enrichment and signaling pathways analysis of the mutations in PLODs and their 45 frequently altered neighbor genes in ccRCC patients.** (**A**) Network of PLODs and their 45 frequently altered neighbor genes was constructed using cBioPortal. (**B**) The functional annotation analysis of PLOD1/2/3 was constructed using ClueGO, a plug-in of Cytoscape. Corrected *p*-value <0.01 was considered statistically significant. (**C**) Functional and pathway enrichment analyses of PLOD1/2/3 were performed using DAVID and visualized in bubble chart. (**D**) Functional and pathway enrichment analyses of 48 involved genes were performed using DAVID and visualized in bubble chart.

We next explored the functional ramifications of PLOD1/2/3 overexpression in ccRCC by investigating the network of interacting genes in *silico* (Figure 7A). Using cBioPortal tools, we constructed a network map for PLOD1/2/3 and the 45 neighboring genes most frequently altered in ccRCC. ClueGO and CluePedia functional annotations revealed a predominantly collagen-focused molecular network (Figure 7B). We also analyzed the GO and KEGG biological classifications of the components of the PLOD functional network using DAVID. In GO analysis, genes in the PLOD1/2/3 network were significantly enriched mainly in lysine degradation pathways, together with oxidation reduction, endoplasmic reticulum, PLOD activity, oxidoreductase activity, L-ascorbic acid binding, carboxylic acid binding, vitamin binding and iron ion binding (Figure 7C, 7D). In the KEGG analysis, genes significantly enriched in the ccRCC dataset compared with normal kidney tissue included lysine degradation, small cell lung cancer, focal adhesion pathways, and extracellular matrix–receptor interaction signaling pathways (Figure 7C, 7D).

**Figure 8:**
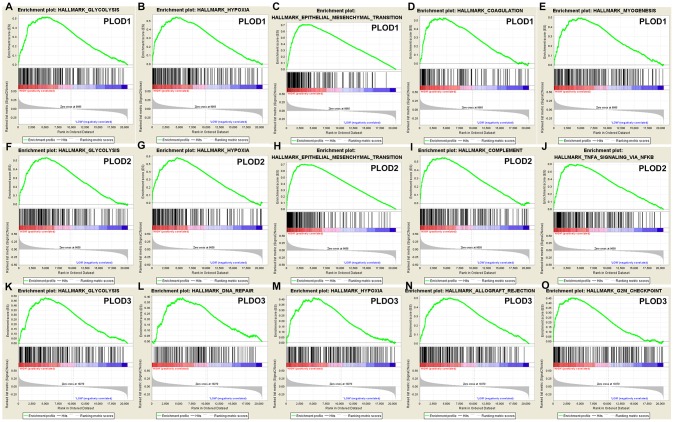
**GSEA was used to perform hallmark signaling analysis in PLOD1/2/3, respectively.** A total of 100 significant genes were obtained from GSEA with positive and negative correlation. The most involved significant pathways included glycolysis, hypoxia, epithelial mesenchymal transition, etc.

Finally, we performed a complementary analysis with GSEA and obtained a total of 100 genes significantly positively and negatively correlated with PLOD1/2/3. As shown in Figure 8, some of the most significantly associated pathways included glycolysis, hypoxia, epithelial–mesenchymal transition, coagulation/ complement, and DNA repair.

## DISCUSSION

Genetic alterations and abnormal epigenetic regulation are known to affect the development and progression of RCC [28]. The functions of PLOD1/2/3 in the synthesis of collagen suggest that defects in its expression and/or regulation may contribute to the aggressive behaviors of many tumors [29, 30]. Although PLODs have been confirmed to play oncogenic roles in many cancers, including breast, lung, and colorectal carcinomas, the prognostic implication of PLOD expression and/or function in ccRCC remained to be elucidated. In this study, we investigated the expression level, mutation rate, biological networks, and prognostic value of PLOD1/2/3 in ccRCC.

More than 30 genetic alterations in the PLOD1 gene have been found to cause Ehlers–Danlos syndrome, a group of disorders that affect the connective tissues that support the skin, bones, blood vessels, and many other organs and tissues [31, 32]. Elevated collagen deposition and cross-linking are also known to contribute to the aggressive behavior of cancer by enhancing invasion, migration, and proliferation, thereby contributing to poor prognosis [33–35]. Significantly increased expression of PLODs has been documented in many carcinomas. For example, the high expression of PLOD1 and PLOD2 observed in esophageal squamous-cell carcinoma is negatively associated with expression of tumor suppressor gene [30]. Gjaltema et al. found that PLOD2 expression was significantly upregulated in breast cancer compared with adjacent normal mammary tissue, and the expression level correlated with poorer PFS [36]. Consistent with this, knockdown of PLOD2 expression in a breast cancer cell line reduced collagen deposition in the primary tumor and reduced tumor growth in a mouse model [11]. In hepatocellular carcinoma, PLOD2 expression correlated with aggressive progression [37]; moreover, PLOD3 was found to be highly expressed in this cancer and may be a promising diagnostic biomarker [29, 38]. In addition, the incidence and growth of hepatocellular cancer was decreased in PLOD2-deficient mice compared with wild-type mice [29]. PLOD2 and PLOD3 were among 54 glycoproteins found to be upregulated in colorectal cancer compared with normal tissue [39]. PLOD2 and another collagen cross-linking enzyme, lysyl oxidase homolog-2, were shown to be epigenetically regulated by the tumor-suppressive microRNAs miR-26a and miR-26b and to promote metastasis in RCC [40]. The latter observation may shed light on the potential association between kidney fibrosis and tumorigenesis [41].

Many growth factor- and cytokine-associated signaling pathways and transcription factors, including HIF-1α and E2Fs, are involved in the transcriptional regulation of PLODs. In cervical carcinoma, hypoxia- and TGF-β-induced PLOD2 expression increases the proliferative, adhesive, and invasive capabilities of cells by promoting the epithelial–mesenchymal transition and formation of focal adhesions [12, 42]. In lung cancer cell lines, ectopic PLOD2 expression rescued the migration and metastasis defects resulting from HIF-1α silencing, and the defects were again reinstated by concomitant inhibition of PLOD2 activity [43]. Gilkes et al. suggested that HIF-1-induced PLOD2 expression plays a critical role in fibrillary collagen formation, tumor stiffness, and metastatic potential in breast cancer [11]. In lung cancer tissue, PLOD2 has been shown to hydroxylate telopeptidyl lysine residues on collagen, which indirectly reduces the levels of lysine aldehyde–derived collagen cross-links [14]. Also in lung cancer cells, elevated PLOD2 expression promotes collagen cross-links, activates the phosphoinositide 3-kinase pathway, and may promote proliferation, invasion, and migration [44]. The results of the GO enrichment analysis of PLOD-neighboring genes in the present study are consistent with this possibility.

Our study is the first to investigate the possible prognostic utility of PLODs in ccRCC. While the preceding discussion illustrates the potential involvement of PLODs in the development of many cancers and human diseases, it is noteworthy that little is known about their involvement in ccRCC. In this study, we demonstrated the significant upregulation of PLODs at the mRNA and protein level in ccRCC and its correlation with disease stage, pathological grade, PFS, and OS. Interestingly, we also showed that genetic mutation of PLOD1/2/3, which is present in ~3% of ccRCC patients, was associated with significantly poorer prognosis compared with wild-type PLOD1/2/3.

There are several limitations to this study. First, the expression of PLOD mRNA was identified as a prognostic biomarker for PFS and OS in this study. However, although PLOD protein levels were highly upregulated in tumor compared with normal kidney tissues, the prognostic implication of this change was not demonstrated. Second, further validation studies and/or prospective cohort studies are needed to verify these findings. Finally, we did not explore the underlying molecular mechanisms by which PLOD upregulation might impact ccRCC behavior, although we did perform *in silico* analyses of PLOD functional networks. Future research will explore the mechanistic differences between the PLOD isoforms in ccRCC and other carcinomas.

In conclusion, our study is the first to demonstrate that PLOD1, PLOD2, and PLOD3 mRNA and protein expression is elevated in ccRCC compared with normal kidney, and that high PLOD1/2/3 mRNA levels predict poor prognosis. These novel findings not only shed light on the molecular alterations in ccRCC but also provide the foundation for further research in this area.

## MATERIALS AND METHODS

### Ethics statement

The study protocol was approved by Fudan University Shanghai Cancer Center (Shanghai, China). Written informed consent was obtained by the online databases from which all patient data were obtained.

### Oncomine data

Transcriptional expression of PLODs in 20 common neoplasms was analyzed using the Oncomine online database (http://www.oncomine.com) [45]. Differentially expressed mRNAs were selected using the cut-off criteria: *p* = 0.01 (Student’s t-test), fold difference in expression 1.5, and differentially expressed gene rank ≤10%.

### Human protein atlas data

Protein expression data in normal and ccRCC tissue samples were obtained from The Human Pathology Atlas project (https://www.proteinatlas.org), which provides immunohistochemical staining data of tissue microarrays for multiple normal tissue types and cancers [46]. Staining intensity, quantity, and location, and associated patient clinical data were available online.

### The cancer genome atlas (TCGA) data

Six RNA-sequencing datasets representing a total of 533 ccRCC specimens and 72 normal kidney tissues with complete clinical data were obtained from the TCGA-KIRC database [47]. Level 3 data were downloaded and processed as described below.

Clinical and pathological parameters of 533 ccRCC patients, specifically age at surgery, gender, tumor laterality, pT stage, pN stage, pM stage, American Joint Committee on Cancer (AJCC) stage, International Society of Urological Pathology (ISUP) grade, were summarized in Supplementary Table 1.

### cBioPortal

The TCGA datasets were analyzed using the cBioPortal resource (http://www.cbioportal.org) [48]. We analyzed the genomic profiles of PLOD1/2/3 for mutations and putative copy-number alterations (GISTIC) and mRNA expression z-scores (RNASeq V2 RSEM) with a threshold of ±1.8. *P* values <0.05 were considered significant in all tests, using built-in Kaplan–Meier analysis method of cBioPortal website to measure survival benefits.

### Functional annotation and pathway analysis

Significantly co-regulated networks of PLOD1/2/3 and their 45 frequently altered neighbor genes were constructed using cBioPortal. Gene Ontology (GO) analysis for biological process, cellular component, and molecular function terms, and Kyoto Encyclopedia of Genes and Genomes (KEGG) pathway analyses for the 48 genes were performed using the Database for Annotation, Visualization and Integrated Discovery (DAVID) functional annotation tool [49]. Data were visualized by constructing bubble charts. Cytoscape (version 3.5) and its plug-ins ClueGO (version 2.5.3) and CluePedia (version 1.5.3) were used to visualize the molecular interaction networks [50, 51].

### Gene set enrichment analysis (GSEA)

The TCGA datasets were analyzed using GSEA software package version 2.10.1. Significantly different pathways were identified with a 1000× permutation test. False discovery-adjusted *p* values were obtained using the Benjamini and Hochberg method [52]. Significant differential expression was defined as an adjusted *p* value of <0.01 and a false discovery rate of <0.25.

### Statistical analysis

Progression-free survival (PFS) was defined as the length of time during and after surgery section of ccRCC that a patient lives without getting worse, and overall survival (OS) was defined as the date of first therapy to the date of death or last follow-up. Survival was analyzed using the Kaplan–Meier method with 95% confidence intervals (95%CI) and the log-rank test. Univariate and multivariate analysis were performed with Cox logistic regression models to identify significantly associated traditional independent variables, including pT stage, pN stage, pM stage, AJCC stage, ISUP grade, and PLOD1/2/3 expression level. X-tile software [53] was used to define the cut-off values to dichotomize patients based on PLOD expression. All tests were two-sided and *p* values <0.05 were considered significant. Statistical analysis and graphical plotting were conducted using R software (version 3.3.2).

### Ethics approval

The Ethics approval and consent to participate of the current study was approved and consented by the ethics committee of Fudan University Shanghai Cancer Center and online databases.

## Supplementary Material

Supplementary Figure

Supplementary Tables
