# Supplementary material for: Procollagen-lysine, 2-oxoglutarate 5-dioxygenases 1, 2, and 3 are potential prognostic indicators in patients with clear cell renal cell carcinoma
Source: Aging (Albany NY). 2019 Aug 25;11(16):6503–21. doi: 10.18632/aging.102206 (PMC6738415; doi:10.18632/aging.102206)
Supplement: Supplementary Figure [file aging-11-102206-s002.pdf]

SUPPLEMENTARY FIGURE

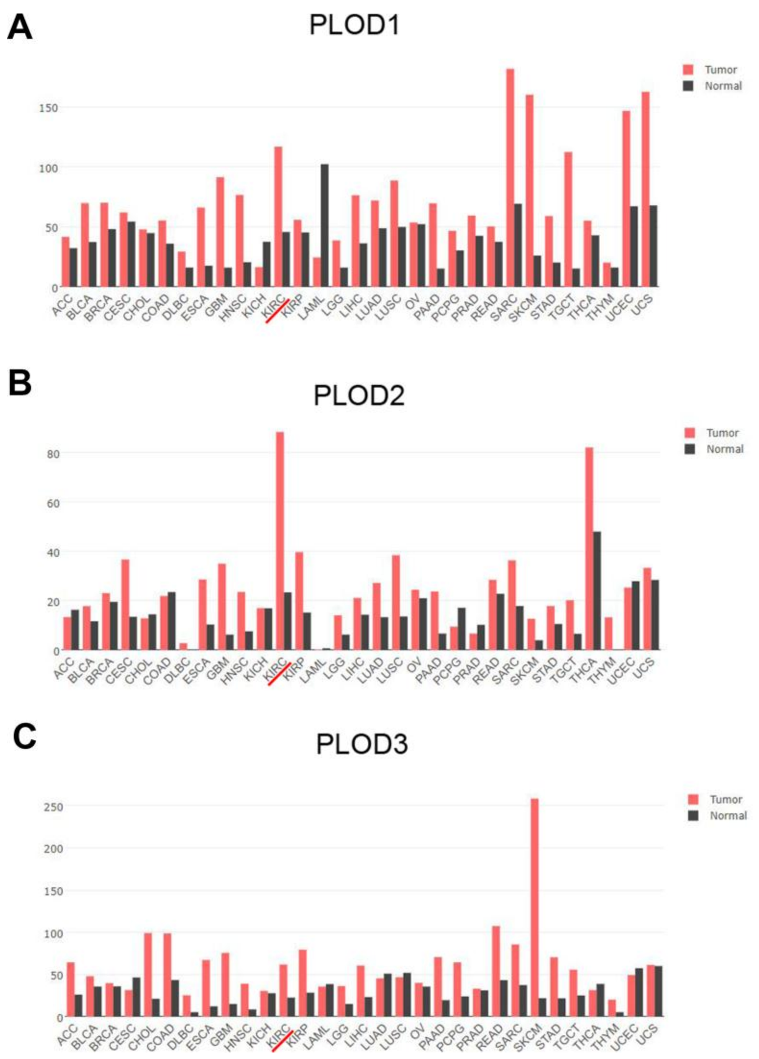

Supplementary Figure 1. PLOD1/2/3 mRNA expression profiles of multi-tumors and corresponding normal tissues.
