# Supplementary material for: Procollagen-lysine, 2-oxoglutarate 5-dioxygenases 1, 2, and 3 are potential prognostic indicators in patients with clear cell renal cell carcinoma
Source: Aging (Albany NY). 2019 Aug 25;11(16):6503–21. doi: 10.18632/aging.102206 (PMC6738415; doi:10.18632/aging.102206)
Supplement: Supplementary Tables [file aging-11-102206-s001.pdf]

SUPPLEMENTARY TABLES

Supplementary Table 1. Clinicopathological characteristics baseline in TCGA-KIRC cohort.

Characteristics	TCGA cohort (N=533)
N (%)	
Age	
< 60 years	245 (46.0)
≥ 60 years	288 (54.0)
Gender	
Male	345 (64.7)
Female	188 (35.3)
Laterality	
Left	251 (47.1)
Right	281 (52.7)
Bilateral	1 (0.2)
pT stage	
T1	273 (51.2)
T2	69 (12.9)
T3	180 (33.8)
T4	11 (2.1)
pN stage	
N0	240 (45.0)
N1	16 (3.0)
Nx	277 (52.0)
pM stage	
M0	422 (79.2)
M1	79 (14.8)
Mx	32 (6.0)
AJCC stage †	
I	267 (50.1)
II	57 (10.7)
III	123 (23.1)
IV	86 (16.1)
ISUP grade	
G1	14 (2.6)
G2	229 (43.0)
G3	206 (38.6)
G4	76 (14.3)
Gx	5 (0.9)

TCGA: the Cancer Genome Atlas, KIRC: kidney clear renal cell carcinoma

†The AJCC staging system is a classification system developed by the American Joint Committee on Cancer for describing the extent of disease progression in cancer patients. It utilizes in part the TNM scoring system: Tumor size, Lymph Nodes affected, Metastases.

Supplementary Table 2. Univariate Cox logistic regression analysis of PFS and OS of PLOD1 in TCGA cohort.

Covariates	PFS			OS		
	HR	95% CI	*p* value	HR	95% CI	*p* value
pT stage (ref. T1-T2)	0.512	0.194–1.351	0.176	0.869	0.504–1.498	0.614
pN stage (ref. N0)	0.871	0.375–2.022	0.748	1.678	1.110–2.535	**0.014**
pM stage (ref. M0)	1.712	0.867–3.380	0.122	1.966	1.357–2.847	**<0.001**
AJCC stage (ref. I-II)	6.743	2.038–22.302	**0.002**	2.321	1.220–4.416	**0.010**
ISUP grade (ref. 1-2)	1.414	0.680–2.944	0.354	1.495	1.033–2.163	**0.033**
PLOD1 expression (ref. low)	3.508	1.537–8.003	**0.003**	1.669	1.132–2.460	**0.010**

PFS: progression-free survival; OS: overall survival; TCGA: the Cancer Genome Atlas.

Supplementary Table 3. Univariate Cox logistic regression analysis of PFS and OS of PLOD2 in TCGA cohort.

Covariates	PFS			OS		
	HR	95% CI	*p* value	HR	95% CI	*p* value
pT stage (ref. T1-T2)	0.484	0.187–1.250	0.134	0.748	0.436–1.285	0.293
pN stage (ref. N0)	1.155	0.579–2.304	0.683	1.562	1.053–2.317	**0.027**
pM stage (ref. M0)	1.591	0.831–3.045	0.161	1.948	1.351–2.808	<**0.001**
AJCC stage (ref. I-II)	5.561	1.750–17.671	**0.004**	2.499	1.333–4.687	**0.004**
ISUP grade (ref. 1-2)	1.949	0.965–3.939	0.063	1.526	1.060–2.196	**0.023**
PLOD2 expression (ref. low)	3.467	1.762–6.823	<**0.001**	2.089	1.489–2.932	<**0.001**

PFS: progression-free survival; OS: overall survival; TCGA: the Cancer Genome Atlas.

Supplementary Table 4. Univariate Cox logistic regression analysis of PFS and OS of PLOD3 in TCGA cohort.

Covariates	PFS			OS		
	HR	95% CI	*p* value	HR	95% CI	*p* value
pT stage (ref. T1-T2)	0.675	0.260–1.752	0.420	0.827	0.481–1.423	0.494
pN stage (ref. N0)	1.688	0.868–3.282	0.123	1.779	1.203–2.629	**0.004**
pM stage (ref. M0)	1.651	0.857–3.182	0.134	2.004	1.384–2.901	<**0.001**
AJCC stage (ref. I-II)	5.424	1.709–17.220	**0.004**	2.513	1.338–4.719	**0.004**
ISUP grade (ref. 1-2)	1.668	0.831–3.349	0.150	1.586	1.103–2.280	**0.013**
PLOD3 expression (ref. low)	2.555	1.428–4.574	**0.002**	1.569	1.146–2.148	**0.005**

PFS: progression-free survival; OS: overall survival; TCGA: the Cancer Genome Atlas.

Supplementary Table 5. Six tumor types with the most significant difference in PLOD 1/2/3 expression besides KIRC.

Gene	Tumor type	PFS *P*-Value	OS *P*-Value
PLOD1	SARC	0.19	**0.014**
	SKCM	0.41	0.77
	UCS	0.13	0.67
	UCEC	0.16	0.84
	TGCT	0.94	0.72
	GBM	0.16	**0.018**
PLOD2	THCA	0.54	0.52
	CESC	0.15	**0.044**
	GBM	0.47	0.43
	KIRP	0.17	0.23
	LUSC	0.49	0.44
	SARC	**0.004**	**0.029**
PLOD3	SKCM	0.27	0.34
	CHOL	0.79	0.31
	COAD	0.14	0.27
	KIRP	0.34	0.69
	READ	0.37	0.051
	SARC	0.49	0.11

SARC: Sarcoma; SKCM: Skin Cutaneous Melanoma; UCS: Uterine Carcinosarcoma; UCEC: Uterine Corpus Endometrial Carcinoma; TGCT: Testicular Germ Cell Tumors; GBM: Glioblastoma multiforme; THCA: Thyroid carcinoma; CESC: Cervical squamous cell carcinoma and endocervical adenocarcinoma; KIRP: Kidney renal papillary cell carcinoma; LUSC: Lung squamous cell carcinoma; CHOL: Cholangiocarcinoma; COAD: Colon adenocarcinoma; READ: Rectum adenocarcinoma Esophageal carcinoma.
